# Long-read sequencing reveals novel structural variation markers for key agronomic and quality traits of food-grade soybean

**DOI:** 10.3389/fpls.2025.1557748

**Published:** 2025-04-08

**Authors:** Zhibo Wang, Kassaye Belay, Joe Paterson, Patrick Bewick, William Singer, Qijian Song, Bo Zhang, Song Li

**Affiliations:** ^1^ School of Plant and Environmental Sciences, Virginia Tech, Blacksburg, VA, United States; ^2^ Graduate Program in Genetics, Bioinformatics and Computational Biology, Virginia Tech, Blacksburg, VA, United States; ^3^ Soybean Genomics and Improvement Laboratory, Beltsville Agricultural Research Center, Beltsville, MD, United States

**Keywords:** long-read sequencing technology, structural variation, gene expression, food-grade soybean, seed weight, plant height, Kunitz trypsin inhibitor

## Abstract

Long read sequencing has been widely used to detect structure variations that are not captured by short read sequencing in plant genomic research. In this study, we described an analysis of whole genome re-sequencing of 29 soybean varieties using nanopore long-read sequencing. The compiled germplasm reflects diverse applications of food-grade soybeans, including soy milk and tofu production, as well as consumption of natto, sprout, and edamame (vegetable soybean). We have identified 365,497 structural variations in these newly re-sequenced genomes and found that the newly identified structural variations are associated with important agronomic traits. These traits include seed weight, flowering time, plant height, oleic acid content, methionine content, and Kunitz trypsin inhibitor content, all of which significantly impact soybean production, quality, and market value. Experimental validation supports the roles of predicted candidate genes and structural variants in these biological processes. Our research provides a new source for rapid marker discovery in soybean and other crop genomes using structural variation and whole genome sequencing.

## Introduction

Soybean is an important crop for animal feed, human consumption and biodiesel production (Vasudevan and Briggs, 2008) and improving genetic gain and nutritional value remains a key priority to meet the ever-growing demand of human population. Exploring and integrating the genomic variants resources provides an opportunity to develop novel tools for genomic innovations, agronomic trait discovery and molecular breeding. Hence, its whole genome sequence plays a critical role as it allows the discovery of genes and their functions, as well as the development of genetic markers for selection.

Advances in next generation sequencing has accelerated the discovery of genomic variations by resequencing of diverse germplasm including wild, landraces and commercial cultivars. These resequencing efforts mostly focused on identification and utilization of SNP markers for trait association, diversity analysis and understanding domestication sweeps. This is largely due to the high prevalence in genome, availability of user-friendly bioinformatics tools detecting them, and their compatibility with genotyping assays for molecular diagnostics. However, SNPs alone do not fully explain the genetic basis of key phenotypic traits in soybean.

Recent research has demonstrated that another type of genetic variants, structural variations (SVs), plays an equally important role in plant evolution and agriculture (Escaramis and Docampo, 2015; Alonge et al., 2020; Liu et al., 2020; Liao et al., 2021). SVs generally refer to DNA regions of at least 50 bp in size, including deletions, insertions, duplications, and chromosomal rearrangements. These variations have been linked to numerous agriculturally significant traits, such as disease resistance in soybean (Cook et al., 2012), stress response in potato (Hardigan et al., 2016), boron toxicity in barley (Sutton et al., 2007), and flowering time in wheat (Wurschum et al., 2015). Despite their importance, the identification of SVs has lagged behind due to lack of robust computational methods and reliable long-read sequencing technologies for accurate detection and characterization. Thus, genetic improvement efforts have been constrained by limited knowledge of structural genomic variations and their contributions to key agronomic traits.

The third generation long-read sequencing technology has been widely adopted in the research community, since it can facilitate the identification of SVs that are unable to be captured by short-read sequencing technology (Liu et al., 2020; Chawla et al., 2021; Hufford et al., 2021). In soybean, a limited number of studies have identified SVs by comparing resequencing data of wild soybeans and also comparing with major sub-populations (Xie et al., 2019). Additionally, some studies were performed to elucidate the association between SVs with phenotypes. The Wm82 genome assembly (v2), widely used as a reference, was initially built on short reads and Sanger sequencing. Gap-filling in later versions (v3 and v4) incorporated PacBio-based BAC assemblies, and v4 also integrated long-read sequencing data (Valliyodan et al., 2019). These rich genomic resources offer an unprecedented opportunity to explore untapped genetic diversity in publicly available soybean genomes.

In this study, we aimed to bridge the knowledge gap regarding the role of SVs in food-grade soybean by leveraging long-read sequencing technology. We conducted whole-genome resequencing of 29 soybean varieties using nanopore long-read sequencing. These varieties represent diverse applications, such as livestock feed, soy milk, tofu, natto, sprouts, and edamame. Our primary objective was to systematically identify and characterize SVs associated with key agronomic traits and assess their potential functional impacts. We identified 365,497 structural variations associated with key agronomic traits, including seed weight, flowering time, plant height, oleic acid content, methionine content, and trypsin inhibitor content, all critical to soybean production and quality. Experimental validation confirmed the roles of candidate genes and structural variants, providing a valuable resource for advancing marker-assisted selection and accelerating trait improvement in soybean breeding programs.

## Methods and materials

### Plant material

We chose 29 Glycine genotypes (**Supplementary Table S1**) as the sequencing representatives for this study. All the plants were grown in the Kentland farm, Blacksburg, Virginia in the year of 2021.

### DNA isolation for Oxford nanopore technology sequencing

High-molecular-weight DNA was isolated using DNA isolation protocol modified from Alonge et al (Alonge et al., 2020). Five grams of young soybean leaves from 4-6-week-old plants were harvested and frozen by liquid nitrogen. Frozen leaf material was ground to fine powder using a mortar and pestle and transferred to a 50 mL Falcon tube. A total of 15 mL of pre-heated lysis buffer 1.4 M NaCl, 100 mM Tris pH 8.0, 2% CTAB (Hexadecyltrimethylammonium bromide, w/v), 20 mM EDTA, 0.5% Na_2_S_2_O_5_ (w/v), 2% 2-Mercaptoethanol (v/v, added freshly)) was added into the 50 mL Falcon tube. The lysate was incubated for 20 min at 60°C. A 15 ml chloroform/isoamyl alcohol (24:1) was added to the lysate to allow proper separation of the organic phase and aqueous phase and keep DNA protected into the aqueous phase. After centrifuge, a 12 cold isopropyl alcohol was added to precipitate the High-molecular-weight DNA. The eluted DNA was treated by RNase at 37°C for 1 hour to degrade the RNA in the elution. The solution of chloroform/isoamyl alcohol (24:1) was then utilized to remove the RNase. After washing by 70% ethanol twice, the DNA was finally eluted by 1 X TE buffer (10 mM Tris, pH 8.0 and 1 mM EDTA). The gel-electrophoresis, qubit (Thermo Fisher), and tape-station (Agilent) were used to determine the concentration and quality of the DNA.

### Library preparation for ONT sequencing

Two ug of DNA was used to prepare the sequencing library, using the ligation sequencing kit SQK-LSK110 according to the manufacturer’s recommendations. Genomic DNA was subjected to end repair (New England Biolab Inc). After a bead clean-up (Applied Biosystems), sequencing adaptors were then ligated to the end-repaired DNA. Finally, the adaptor ligated DNA was once again subjected to bead cleaning. The DNA library was finally loaded onto an Oxford Nanopore PromethION flow cell for sequencing at Virginia Tech core facility, Genomic Sequencing Center.

### SV calling and sorting

For each of our 29 soybean genotypes selected for this study, ONT sequencing technology was used to generate long reads. Raw reads were base called on a GPU using Oxford Nanopore Technologies’ guppy base caller v. 5.0.11 with parameters –flow cell FLO-PRO002 –kit SQK-LSK110. Raw FASTQ files obtained from a single flow cell were then concatenated into a single file which was used for downstream analyses. Concatenated raw reads were aligned to the Wm82.a4 soybean reference genome (Valliyodan et al., 2019). The Wm82.a4 soybean reference genome is a recently published preprint that improves to the previous Wm82.a2 soybean reference genome. This new reference genome is the most complete and accurate representation of the Wm82.a2 reference genome to date. The reference gene models used in this study, are the accompanying reference gene annotation set. Structural variants were called relative to this reference genome from aligned reads with MINIMAP2 (v2.23, –MD -Y -ax map-ont -t 50) and the resulting alignments were sorted and indexed using samtools (v 1.7, samtools view -bS, samtools sort -@ 20, samtools index -@ 20). To call SVs, we run Sniffles with parameters (sniffles -t 5 -s 20 -r 2000 -q 20 -d 1000 –genotype -l 30 -m, minimum read segment length for consideration = 1000, default = 2000). We chose relaxed parameters compared to the defaults because our samples are inbred cultivars and heterozygosity should therefore be nearly non-existent (Sedlazeck et al., 2018). As is convention, SV labels (insertions, deletions, duplications, inversions, translocations, and inversion-duplications) are defined with respect to this single reference genome and do not necessarily define the underlying mutations causing the genetic variation.

We applied a series of filters using bcftools (Danecek et al., 2021) (bcftools view -i ‘(SVTYPE = “DUP” || SVTYPE = “INS” || SVTYPE = “DEL” || SVTYPE = “INV” || SVTYPE = “INVDUP” || SVTYPE = “TRA”) && ABS(SVLEN) > 49 && ABS(SVLEN) < 15000’) to remove any spurious calls that could affect downstream analyses. Any variants smaller than 50 nucleotides or larger than 15 kb were removed. We only retained deletions, insertions, inversions, duplications, and inversion-duplications for further analyses. We discarded unresolved breakpoints (SVTYPE=BND) as well as other complex types such as DEL/INV, DUP/INS, and INV/INVDUP variants. We annotated all filtered structural variants based on their overlap with various gene features using R packages; GenomicFeatures, GenomicRanges and ChIPseeker (Lawrence et al., 2013; Yu et al., 2015). We retrieved the positions of the gene models for Williams82 assembly 4 from Phytozome and determined the location of peaks for each SVs in terms of genomic features of the following genic features: Promoter (<=1kb), Promoter (1-2kb), Promoter (2-3kb), Downstream (<= 300), and intergenic (3-15kb).

### Genomic validation

The selected structural variation candidates were validated by PCR using the corresponding genomic DNA as the template, where the WM82 genomic DNA served as the control. The PCRs were performed by *Taq* 2X Master Mix (New England Biolab Inc) according to the manufacturer’s instructions. Oligo primers are listed in **Supplementary Table S6**.

### RNA isolation and real-time PCR

All RNA was extracted from soybean tissues using TRIzol reagent (Thermo Fisher Scientific) according to the manufacturer’s instructions and the method described by Wang et al (Wang Z. et al., 2023). DNA residue was eliminated by treatment with UltraPure DNase I (Thermo Fisher Scientific). The integrity and quantity of total RNA were determined by electrophoresis in 1% agarose gel and a NanoDrop ND-1000 spectrophotometer (NanoDrop Technologies, Wilmington, DE). cDNA synthesis was performed using the SuperScript III First-Strand RT-PCR Kit (Thermo Fisher Scientific) with an oligo-dT primer based on the manufacturer’s instructions. Real-time PCR was conducted with cDNA as the template using the Quantitect SYBR Green PCR kit (Qiagen) according to the manufacturer’s protocol. Oligo primers are listed in **Supplementary Table S1**. The soybean ELF1B gene was used as a reference gene, and data is presented as ΔCT.

### HPLC method to quantify kunitz trypsin inhibitor

The HPLC method for quantifying KTI was conducted in accordance with a previously established protocol (Rosso et al., 2018). In brief, 10 mg of finely ground soybean seed powder was mixed with 1.5 mL of 0.1 M sodium acetate buffer (pH 4.5). Samples were vortexed and shaken for 1 h at room temperature. Following vigorous vortexing and a 1-hour incubation at room temperature, the sample underwent centrifugation at 12,000 rpm for 15 minutes. Subsequently, 1 mL of the supernatant was filtered through a syringe using an IC Millex-LG 13-mm mounted 0.2-mm low protein binding hydrophilic Millipore (polytetrafluoroethylene [PTFE]) membrane filter (Millipore Ireland).

The KTI in solution was then separated using an Agilent 1260 Infinity series (Agilent Technologies) equipped with a guard column (4.6 x 5 mm) packed with POROS R2 10-mm Self Pack Media and a Poros R2/H perfusion analytical column (2.1 x 100 mm, 10 µm). The mobile Phase A comprised 0.01% (v/v) trifluoroacetic acid in Milli-Q water, while mobile Phase B consisted of 0.085% (v/v) trifluoroacetic acid in acetonitrile. The injection volume was 10 µL, and the detection wavelength was set at 220 nm.

## Results

### Distribution and characterization of structural variants across soybean genome

**Figure 1:**
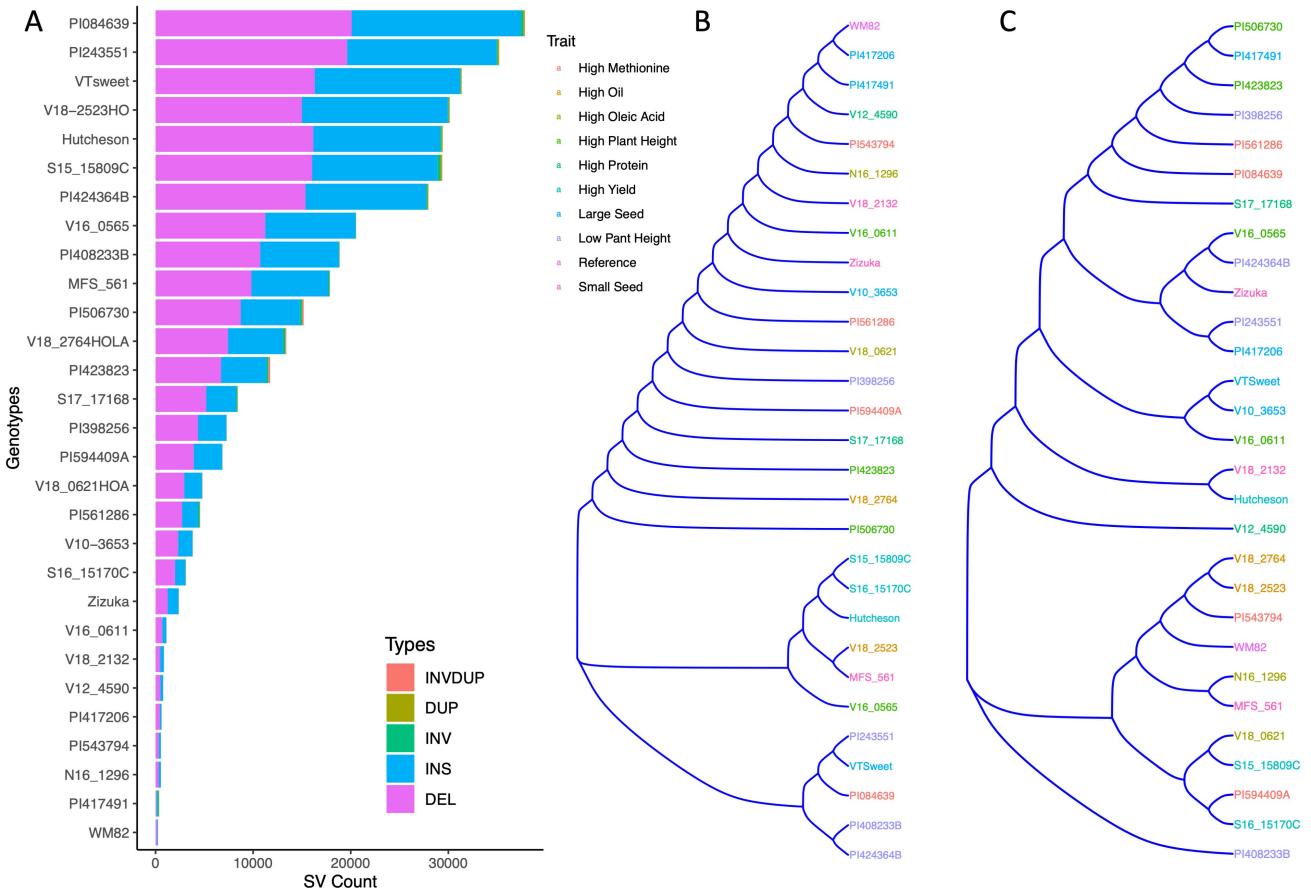
The clustering of 29 soybean/edamame genotypes based on their structural variant presence/absence matrix. **(A)** Stacked bar graph showed structural variant numbers and types from 29 soybean genotypes. **(B)** Hierarchical clustering dendrogram of structural variant presence/absence matrix across 29 soybean genotypes, with colors corresponding to phenotypic traits. **(C)** A SNP-based phylogenetic tree of 29 varieties using 6K SNPs, with colors corresponding to their respective phenotypic traits.

Our objective was to assess the presence, distribution, and the phenotypic effects of large-scale structural variants (> 50bp indels) (Mahmoud et al., 2019; Karaoglanoglu et al., 2020) in soybean genotypes. To achieve this goal, we applied Oxford Nanopore Long-Read Sequencing Technology (ONT) to sequence 28 representative genotypes of both grain-type soybean and vegetable soybean (edamame) as well as the reference variety, William 82 (W82, **Supplementary Table S1**). This germplasm compilation includes grain-type soybeans such as Hutcheson and V12-4590 used in livestock feeding, food-grade soybeans like zizuka (natto), V10-3653 (soy milk and tofu producing variety), MFS-561 (sprout variety), and vegetable soybeans (edamame) including VT-Sweet and V16-0565 (Escamilla et al., 2019; Zhu et al., 2020; Zhang et al., 2021; Williams et al., 2022). These genotypes feature diverse traits such as high methionine content, high yield, varying seed protein levels, distinct plant heights (both high and low), and high oleic acid content, and different seed weights (**Figure 1**, **Supplementary Table S1**).Our study doubled the number of soybean genotypes that were re-sequenced by a long-reading sequencing approach (Liu et al., 2020). For the 29 soybean genotypes, we collected a total of 5.6 Tb of long-read raw sequencing data with an average of 30X genome sequence coverage and an average read length N50 of 11.1 kbp. The recently published Wm82.a4.v1 soybean reference genome (Valliyodan et al., 2019) was utilized to align raw sequencing reads using minimap2 (Li, 2018). The resulting aligned reads were sorted and indexed using samtools (Li, 2011) and structural variants were called with Sniffles (Sedlazeck et al., 2018). Called structural variants were then filtered (defined in this study as >50 bp and < 15,000 bp) and all SV calls for all 29 genotypes were merged. A total of 365,497 SVs were identified and has been uploaded for widespread use within the soybean community as a **Supplementary Dataset**.

**Figure 2:**
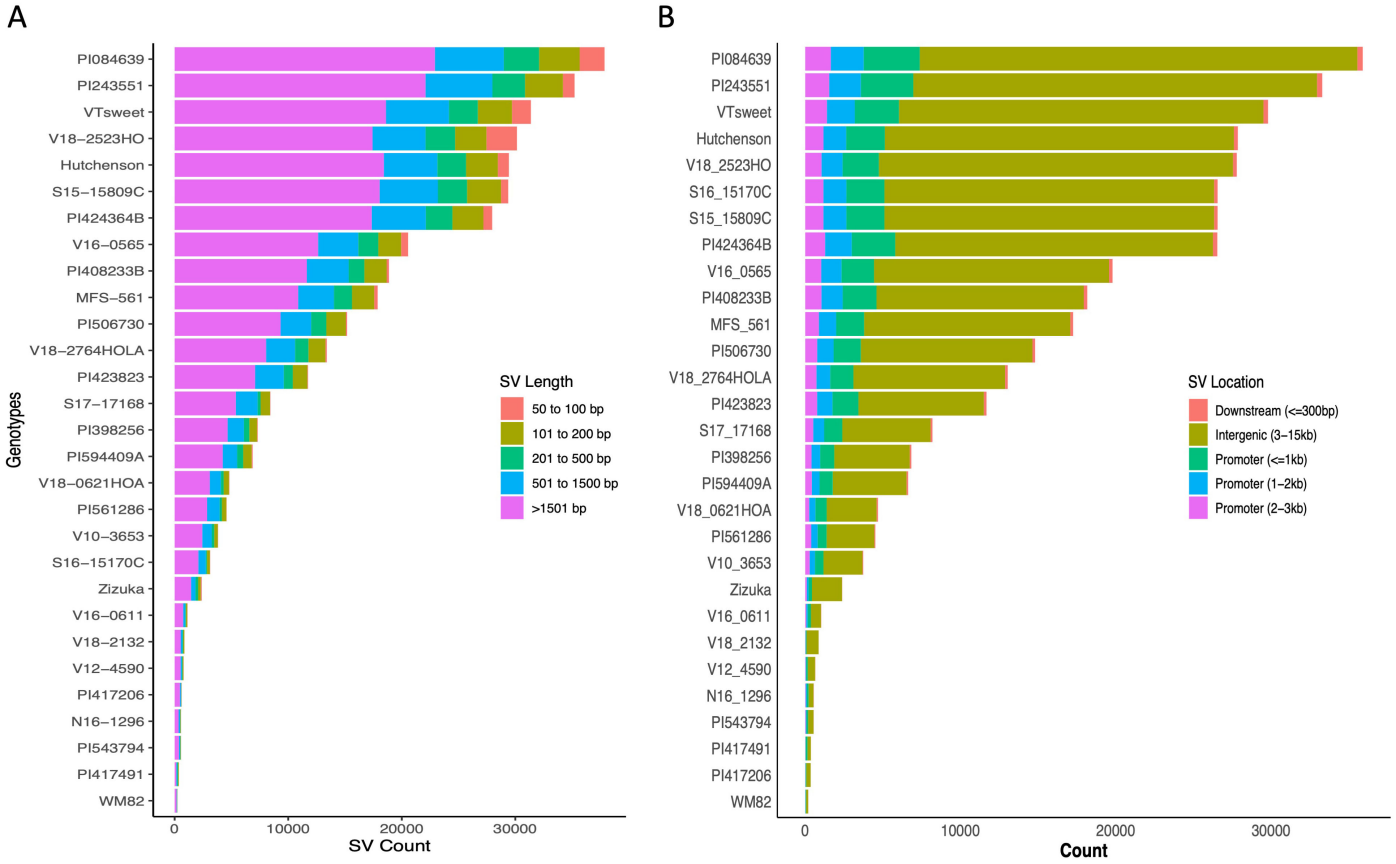
The distribution of SVs across 29 soybean genotypes. **(A)** Stacked bar graph showed structural variant length distributions, **(B)** Stacked bar plot for structural variant distributions across different genomic regions.

These 29 soybean genotypes had SVs between 253 (W82) and 37,863 (PI084639). PI084639, PI243551, V18-2523HO, and VT Sweet, carried the most structural variation relative to the WM82 reference genome (**Supplementary Table S1**). Insertions and deletions were the most common SV types in all genotypes, accounting for more than 95% of the SVs (**Supplementary Table S1**, **Figure 1A**). We also found hundreds of inversions, duplications, and translocations among the accessions (**Supplementary Table S1**, **Figure 1A**). Cluster analysis based on the SV presence/absence matrix showed that the genotypes were clustered into three major groups (**Figure 1B**). Interestingly, genotypes sharing similar representative traits did not form clusters, except for the notable observation that the genotypes we specifically chose (Hutcheson, S16-15170C, and S15-15809C) for high yield were found to cluster together (**Figure 1B**). The two accessions selected as the representatives of low plant height were clustered together but PI424364B had a much greater number of SVs as compared to PI408233B (**Supplementary Table S1**, **Figures 1A, B**). In addition, the same 29 varieties were genotyped using 6K beadchip assay with 6K SNPs to generate a SNP-based phylogenetic tree (**Figure 1C**), which showed a different relatedness as compared to the tree based on SV presence/absence. This result suggests that genetic relatedness of soybean varieties based on structural variation represents a significant deviation from that deduced from SNPs. We further evaluated SVs’ distributions based on their length and location. The length distribution of SVs showed that majority of SVs (61.3%) had the length ranging at 501-1500 bp, followed by 17.6%, 9.9%, 8.0%, 3.2% of SVs within 50-100 bp length, 101 to 200 bp, 201-500 bp, and above 1501 bp, respectively, (**Figure 2A**, **Supplementary Table S2**).

### Impact of structural variants on kunitz trypsin inhibitor levels in soybean seeds

Compared to the SNPs, SVs can cause large-scale perturbations of cis-regulatory regions and are therefore more likely to quantitatively change gene expression and alter related agronomic phenotypes (Saxena et al., 2014; Alonge et al., 2020). For instance, a tandem triplication over the AMTE1 genes is reported to be associated with aluminum resistance in maize (Maron et al., 2013) and an insertion of Ty1/copia-like retrotransposon disrupted the expression level of E4 and caused insensitivity under long day conditions in soybean (Liu et al., 2008). In our analysis, we found that 77% of the total SVs (285, 760 structural variants) are located within intergenic 3-15 kb region (**Figure 2B**, **Supplementary Table S3**). To confirm whether our candidate SVs affected gene expression, and more importantly, on plant phenotypes, we performed experimental validations and statistical analyses to verify the functions of the identified SVs.

**Figure 3:**
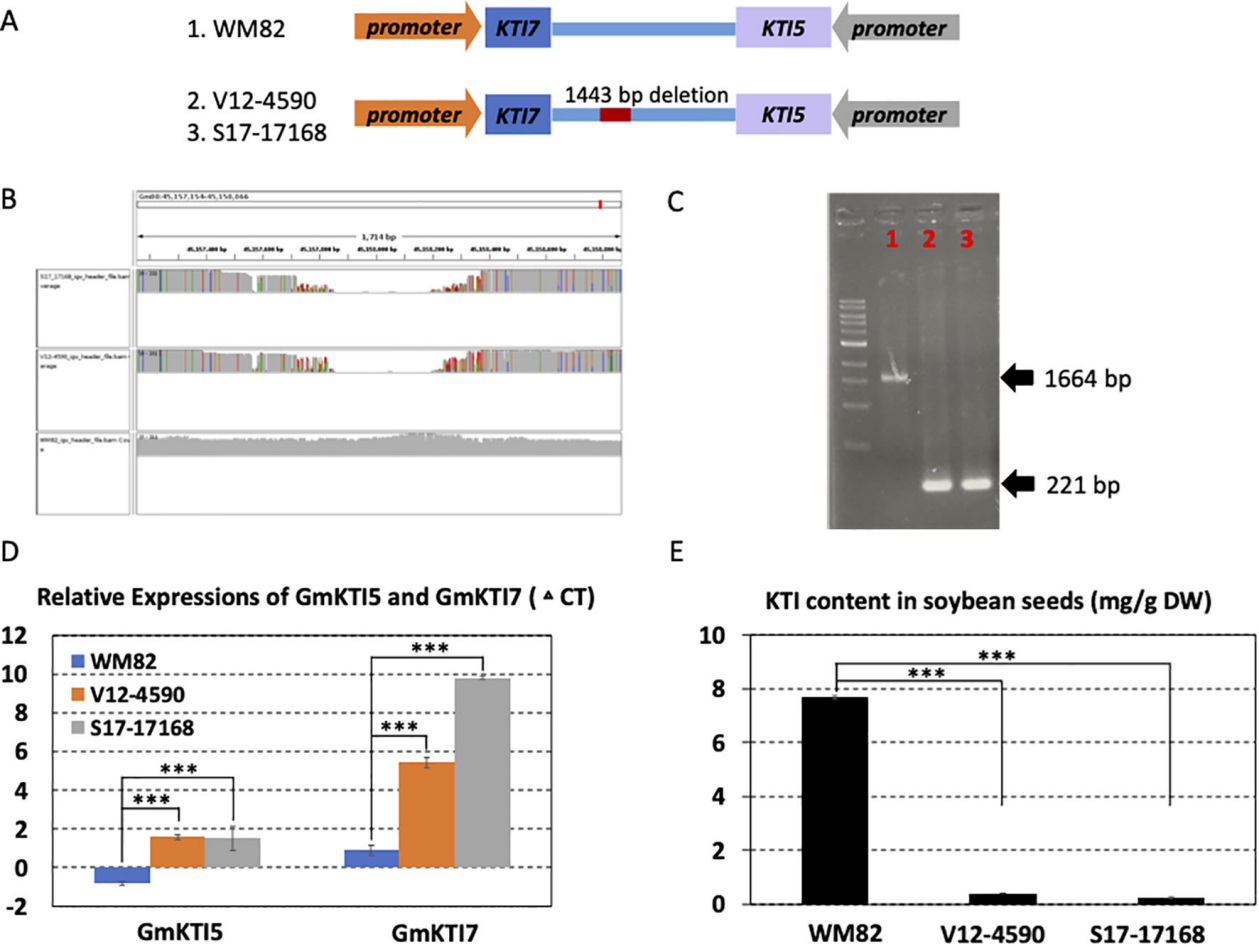
A structural deletion (1443 bp) was found to locate between two KTI genes (Gm08g342200 and Gm08g342300); validated to lower the expression levels of these two genes and reduce the KTI content in the soybean seeds. **(A)** The scheme displayed the location of this structural deletion. The orientations of two genes were opposite, and structural deletion was located at 3’ UTR of these two genes. **(B)** 1443 bp deletion was detected in two lines, V12-4590 and S17-17168 other than William 82 (WM82). **(C)** Agarose gel image of PCR products spanning the deletion polymorphism. M:1 kb ladder; 1: WM82; 2: V12-4590; 3: S17-17168. As expected, WM82 exhibits 1664 bp PCR product, whereas the other two genotypes all show a 221 bp amplicon. **(D)** Real-time PCR was utilized to evaluate the expression levels of Gm08g342200 (KTI 7) and Gm08g342300 (KTI 5) in the seeds of three genotypes. With the structural deletion, the expressions of these two genes in the seeds of V12-4590 and S17-17168 dramatically declined as compared with WM82. **(E)** The KTI contents in the seeds of WM82, V12-4590 and S17-17168 were assessed by HPLC. Consistent with the expression result, the KTI content in the seeds of V12-4590 and S17-17168 was dramatically less than that in the seed of WM82.

In light of previous findings associating QTLs, SNPs, and gene models with seed quality and agronomic performance in soybeans, our study focused on SVs derived from the current research (Luo et al., 2022; Wang et al., 2022; Han et al., 2024). We specifically selected an SV located within a QTL region known to impact soybean seed Kunitz trypsin inhibitor (KTI) for functional validation (Rosso et al., 2021). Soybean meal provides an excellent source of protein in animal feed since it is rich in amino acids with a high nutritional profile. However, the digestibility of soy protein can be severely impacted by KTI, which can restrain the function of trypsin, a critical enzyme that breaks down proteins in the digestive tract (Wang Z. et al., 2023). Traditional heating processes used in soybean meal production deactivate KTI, yet this method not only reduces the meal’s nutritional value due to amino acid degradation but also escalates energy costs by 25%. Raising low-KTI or KTI-free soybeans on farms creates a unique market opportunity for integrated crop and livestock farmers, increasing their farm’s profitability. A major QTL at chromosome 8 in the soybean genome was reported to harbor 13 KTI homologue genes and associate with low KTI in the mapping population (Rosso et al., 2021). The present study found a deletion of 1443 bp in the downstream sequence of two KTI genes (Gm08g342200 (KTI7), Gm08g342300 (KTI5)) in the two inbred lines, V12-4590 and S17-17168 (**Figure 3A**), from Virginia and Arkansas, respectively. The deleted SV was visualized by integrative genomics viewer (IGV) and PCR using the genomic DNAs as templates (**Figures 3B, C**). The real-time PCR showed that the expressions of the two genes were both reduced in the seeds of these two lines in contrast with those in WM82 (**Figure 3D**). The seed KTI content in the two lines was significantly lower than that in WM82 (**Figure 3E**). Here, V12-4590, also names by VT Barrack, was purposely bred as a low KTI soybean variety while S17-17168 has not been reported to be a low KTI variety. Our results revealed a new genetic variation regulating the KTI genes’ expressions in the soybean seeds.

### Impact of structural variants on key agronomic traits in soybeans

Despite significant advancements, several unexplored facets persist within soybean genetics, where structural variants (SVs) potentially wield considerable influence. In order to harness the potential of the SVs called by present study, we employed Chi-squared test followed by Benjamini-Hochberg adjusted p-value thresholds to pinpoint candidate genes associated with six key traits crucial in the soybean industry, including (1) protein content, (2) oleic acid content, (3) methionine content, (4) seed weight, (5) plant height, and (6) flower color (**Supplementary Table S4**, **Supplementary Table S5**). From this pool of traits and genes, we chose two candidate gene models for further validation.

**Figure 4:**
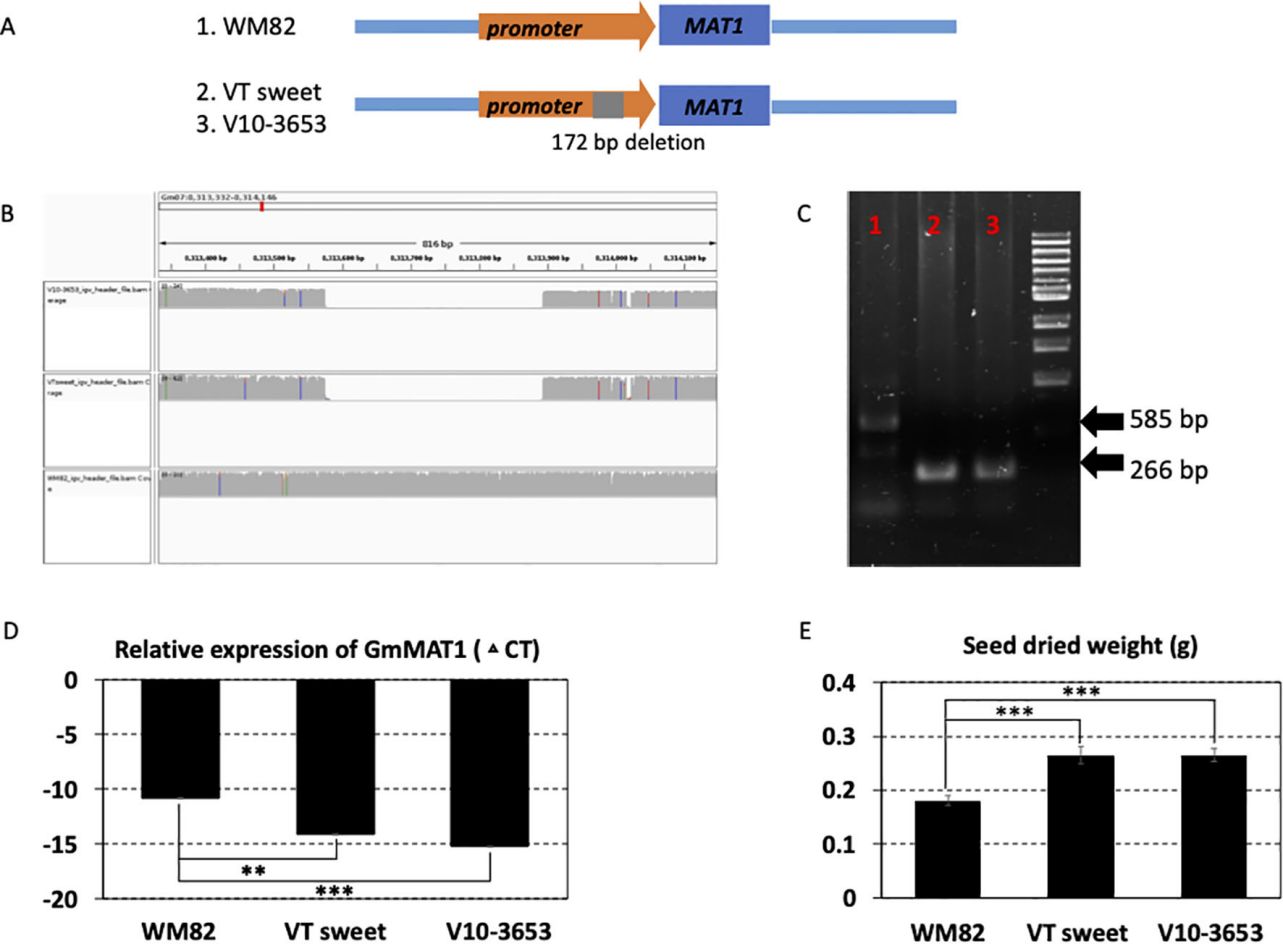
A structural deletion (319 bp) was found to locate at the promoter region of MAT1 (Gm07g090400); validated to enhance its expression level and the size of the soybean seeds. **(A)** The scheme displayed the location of this structural deletion. **(B)** 319 bp deletion was detected in two accessions, VT sweet and V10-3653, other than William 82 (WM82). **(C)** Agarose gel image of PCR products spanning the deletion polymorphism. M:1 kb ladder; 1: WM82; 2: VT sweet; 3: V10-3653. As expected, WM82 exhibits a 585 bp PCR product, whereas the other two genotypes all show a 266 bp amplicon. **(D)** Real-time PCR was utilized to evaluate the expression level of MAT1 in the seeds of three genotypes. With the structural deletion, the gene’s expression in the seeds of VT sweet and V10-3653 dramatically increased in comparison with WM82. **(E)** The seed size of WM82, V10-3653 and VT sweet were assessed by the seed weight, where the seed weight of WM82 was significantly lower than V10-3653 and VT sweet seeds.

A substantial increase in seed size was a major feature of soybean domestication, especially for the vegetable soybean, edamame. According to the annotation in Soybase (Severin et al., 2010), the maturation-associated protein 1 (MAT1, Gm07g090400) gene was associated with seed size. Our results found a 319 bp deletion in the promoter region (-448 bp to TSS) of MAT1 in two food grade soybean varieties, V10-3653 (food grade soybean) and V16-0524 (VT sweet, the edamame variety) (**Figure 4A**). The statistical analysis displayed that the SV locating with MAT1 has a p-value of 1.26E-02, showing a significant association with seed size of soybean seeds (**Supplementary Table S4**). The deleted SV was visualized by IGV and confirmed by PCR using the genomic DNAs as templates (**Figures 4B, C**). With the deletion, the expression of MAT1 gene in the seeds of these two varieties was much higher than that in WM82 (**Figure 4D**). Consistently, the seed weights of the two lines were also much higher than the WM82 seed (**Figure 4E**).

**Figure 5:**
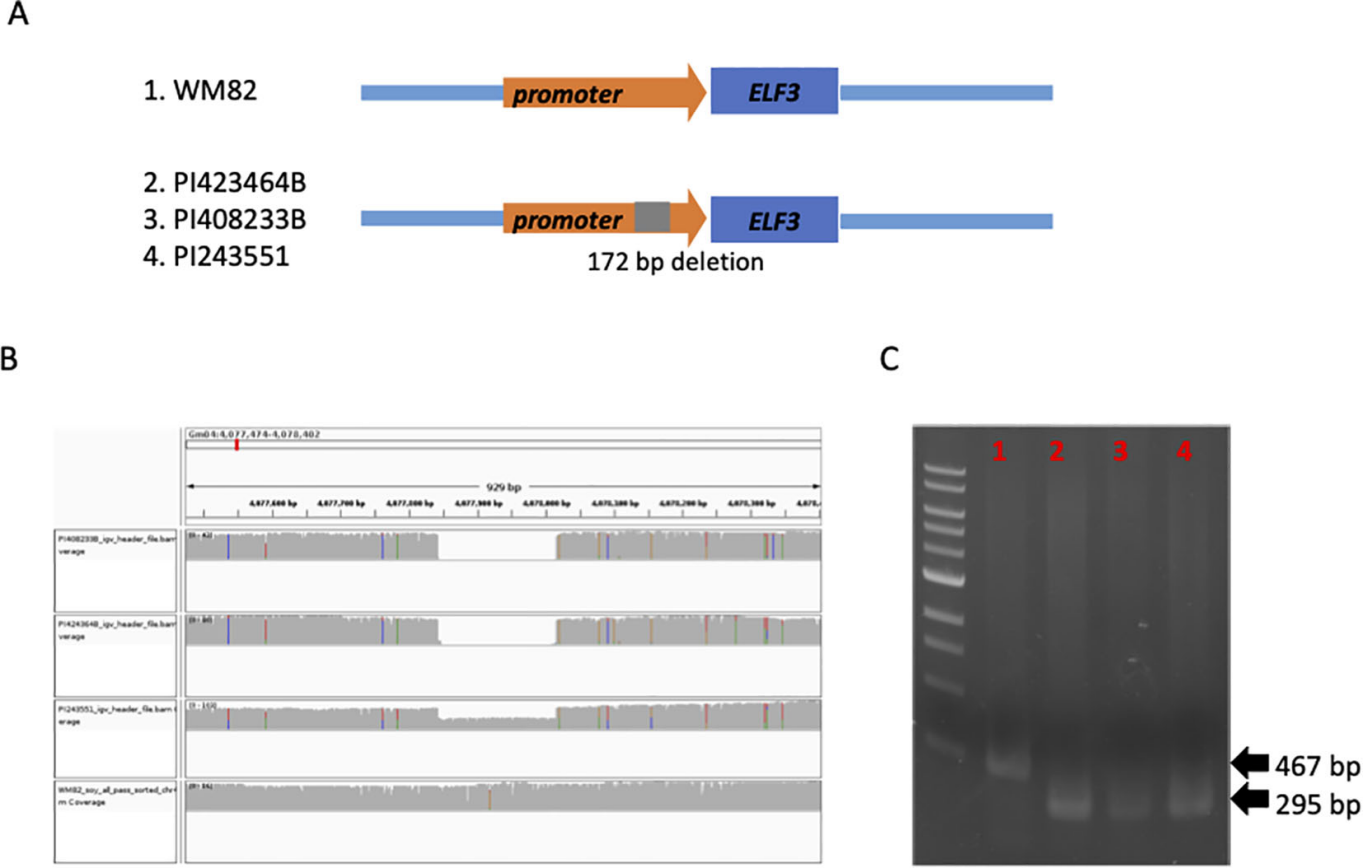
A structural deletion (172 bp) was found to locate at the promoter region of ELF3 (Gm04g050200), which might be associated with flowering time and maturity of soybean plants. **(A)** The scheme displayed the location of this structural deletion. **(B)** 172 bp deletion was detected in two accessions, PI423464B, PI408233B, PI243551, but not WM82. **(C)** Agarose gel image of PCR products spanning the deletion polymorphism. M:1 kb ladder; 1: WM82; 2: PI423464B; 3: PI408233B; 4: PI243551. As expected, WM82 exhibits a 467 bp PCR product, whereas the other three genotypes all show a 295 bp amplicon.

The optimal height for current commercial soybean cultivar contributes to higher yields through improved resistance to lodging, with shorter or taller stands leading to yield reductions (Yang et al., 2021). Here, we found a deletion of 173 bp in the promoter region (-3688 bp to TSS) of EARLY FLOWERING 3 (ELF3, Gm04g050200) gene in three low plant height accessions including PI423464B, PI408233B, and PI243551 (**Figure 5A**). The statistical analysis showed that the SV locating at the promoter region of ELF3 has a p-value of 3.39E-02, suggesting a marginally significant effect on the plant height of soybean plants (**Supplementary Table S4**). All the three accessions belong to the maturity group IV. The presence of the SV was confirmed by IGV and PCR using the genomic DNAs as templates (**Figures 5B, C**). ELF3 was reported as a core component of the circadian clock in the evening complex (Bu et al., 2021). Hence, we speculated the alteration on the expression of ELF3 might be responsible for the early flowering and relatively low plant height of these accessions. The regulating molecular mechanism through the deletion will be studied for comprehensive understanding of the functions of ELF3 in the circadian clock and soybean maturity.

In conclusion, by integrating ONT to re-sequence 29 genotypes of grain type soybean and edamame, we identified novel structure variations that have the potential to reveal the basic genetic mechanism associating with important agronomic traits in soybean. It is fundamental to uncover the mechanism underlying these complex traits. These SVs can be also applied to molecular breeding via CRISPR/Cas9-mediated gene editing and to develop markers for soybean breeding selections. Our results provide a new pipeline for understanding basic genetics and rapid marker discovery in crop genomes using structural variation and whole genome sequencing.

### Data reuse potential

Our study significantly expanded the number of soybean genotypes re-sequenced using a long-read sequencing approach, contributing 12 PI accessions, 10 breeding lines from multiple states, and 7 commercial cultivars. These genotypes exhibit a wide range of traits, including high methionine content, high yield, varying seed protein levels, distinct plant heights (both tall and short), high oleic acid content, and different seed weights (**Figure 1**, **Supplementary Table S1**).

For the 29 soybean genotypes, the sequencing data is of high quality. We collected a total of 5.6 Tb of long-read raw sequencing data, with an average genome sequence coverage of 30X and an average read length N50 of 11.1 kbp. The raw data has been deposited in NCBI and is available for further use, such as pan-genome assembly, by other groups for either breeding or basic research purposes.

## Discussion

### SV impacts soybean agronomic trait by regulating gene expression

As a vital source of food, protein, and oil, the soybean genome serves as a crucial tool for breeders and scientists. It facilitates the discovery of genes, their positions, and functions, as well as the development of marker collections and high-resolution genetic maps. However, current soybean genome sequencing predominantly relies on short-read sequencing technology, which struggles to reliably identify structural variants (SVs). Decades of research have demonstrated that SVs—including large deletions, insertions, duplications, and chromosomal rearrangements—play significant roles in plant evolution and agriculture, influencing key traits such as shoot architecture, flowering time, fruit size, and stress resistance (Alonge et al., 2020; Athiyannan et al., 2022). Consequently, most SVs in soybean remain poorly characterized, leaving their molecular and phenotypic impacts largely unexplored. The recent adoption of long-read sequencing technology offers a major advantage. This technology can identify novel SVs that are often missed by short-read sequencing, providing deeper insights into the soybean genomic landscape. For example, the Wm82 genome assembly (version 4) incorporated long-read sequencing data to address gaps present in its version 2, which relied on short-read and Sanger sequencing (Valliyodan et al., 2019). Furthermore, by utilizing long-read sequencing, long-range scaffolding, and advanced bioinformatics algorithms, telomere-to-telomere (T2T) genome assemblies of soybean accession Wm82 and the southern US accession Lee (PI 548656) were recently completed (Garg et al., 2023; Wang L. et al., 2023). These assemblies resolve gaps that previously hindered the investigation of complex genomic regions, such as centromeres and telomeres (Wang L. et al., 2023). The near-gapless assemblies offer a comprehensive and accurate representation of the soybean genome, encompassing its most complex and highly repetitive regions—such as telomeres, centromeres, and nucleolar organizing regions. These assemblies have significant implications for downstream genome-based studies, including evolutionary analyses and the identification of genes or variations linked to specific traits (Garg et al., 2023).

In this study, we utilized nanopore long-read sequencing to resequence 29 soybean varieties, representing a diverse range of applications, including livestock feed, soy milk and tofu production, natto, sprouts, and vegetable soybeans (edamame). We identified 365,497 structural variations in these newly re-sequenced genomes. Interestingly, 77% of the identified SVs were located in intergenic regions (**Figure 2B**), likely due to the fact that intergenic regions are non-protein-coding and constitute the majority of the genome. Similarly, in pigs, many SVs have been found in intergenic and intronic regions. In Theobroma cacao (the chocolate tree), most SVs are also located in intergenic regions. Furthermore, in Tibetans, nearly all population-stratified SVs were identified within introns or intergenic regions (Du et al., 2021; Hamala et al., 2021; Quan et al., 2021).

Cluster analysis based on the SV presence/absence matrix revealed that the genotypes grouped into three major clusters (**Figure 1B**). In contrast, the phylogenetic tree generated from a 6K SNP beadchip assay (**Figure 1C**) revealed a different genetic relatedness pattern than the SV-based tree. This suggests that structural variation provides distinct insights into genetic relationships, which SNP data alone cannot capture. Consequently, SNPs may not fully reflect the evolution, domestication, or the underlying genomic basis of key agronomic traits in soybeans. The soybean genome is known to contain many homologous regions due to ancient duplication. One hypothesis is that the SVs identified in this study could be false positives if they are located within these homologous regions. Precisely determining these false positives using computational tools requires specialized methodologies to account for ancient genome duplications. In our paper, we focused on validating selected SVs using independent PCR assays, which support the conclusion that these SVs are not false positives from the computational analysis.

Moreover, compared to single nucleotide polymorphisms (SNPs), SVs can induce more profound changes by disrupting cis-regulatory regions, making them more likely to affect gene expression and phenotypic outcomes. SVs can also directly alter gene expression by modifying gene copy number. For example, most soybean cyst nematode (SCN)-resistant soybeans carry a common resistance locus (*Rhg1*), and different copy numbers at the *Rhg1* locus confer varying levels of resistance to SCN (Cook et al., 2012).

In this study, we also identified structural variations (SVs) that can affect gene expression. Specifically, we discovered a 1443 bp deletion downstream of two KTI genes (*Gm08g342200* (KTI7) and *Gm08g342300* (KTI5)) in the soybean varieties V12-4590 and S17-17168 (**Figure 3A**). This structural variation (SV) is located within a QTL region known to influence KTI content in soybean seeds. Real-time PCR analysis revealed that the expression of these two genes was significantly reduced in the seeds of V12-4590 and S17-17168 compared to WM82 (**Figure 3D**), and their seed KTI content was also substantially lower (**Figure 3E**). Our findings uncover a new genetic variation that regulates KTI gene expression in soybean seeds. Interestingly, since KTI is a protein that requires specialized chemical methods to quantify and cannot be easily measured using NIR in breeding programs, the low KTI content in S17-17168 was unknown prior to re-sequencing. This underscores the potential of re-sequencing technology in uncovering phenotypes that require specialized evaluation methods. Moreover, since protein proteases play an active role in seed development and subsequent germination (Martinez et al., 2019), the content and activity of protease inhibitors have also been widely reported to be involved in these processes. The function of this newly identified SV warrants further investigation, implying the potential significance of SVs in soybean seed development.

### SV plays a crucial role in regulating soybean plant height, a key developmental trait

Additionally, in this study, we identified a structural variant (SV) associated with plant height, an important agronomic, developmental, and geographical adaptive trait that is generally linked to plant maturity. Specifically, we discovered a structural deletion in the promoter region of the ELF3 gene, which may be the genetic cause of the reduced plant height observed in soybean accessions PI423464B, PI408233B, and PI243551 (**Figure 5**). ELF3 functions as a core component of the circadian clock within the evening complex, interacting with E1, LUX, and ELF4 to regulate flowering time, and, consequently, plant growth and developmental transitions (Bu et al., 2021). Our finding here suggests a potential mechanism in which the newly identified SV may enhance ELF3 expression, leading to the suppression of the downstream E1 gene, ultimately promoting early flowering and reducing plant height in these accessions. However, further experimental validation is required to confirm this hypothesis.

Understanding soybean plant height is crucial for field management, as optimal height in commercial cultivars enhances resistance to lodging and maximizes yield, whereas excessively short or tall plants can negatively impact productivity. This trait is regulated by multiple genetic variants, with Dt1 (Determinacy 1) on chromosome 19 playing a key role (Liu et al., 2010). Different alleles within Dt1 significantly influence plant height, with taller varieties often carrying specific dt1-t alleles that result in a determinate stem type. Additionally, genetic variants in the E genes (E1, E2, E3, and E4), which control flowering time and maturity, have also been reported to affect soybean plant height (Jiang et al., 2014). While previous studies have largely focused on SNPs and small indels in these genes, SVs have not yet been explored as potential regulators of soybean plant height. Leveraging current genetic knowledge, particularly insights from Dt, E, and ELF genes, and systematically searching for SVs in relation to these loci could provide a valuable resource for advancing our fundamental understanding of plant height and development regulation. This knowledge could, in turn, contribute to improving soybean breeding strategies and optimizing agricultural production.

### The identification of SVs is crucial for the development of food-grade soybean cultivars

In addition to being a rich source of protein and oil for livestock and poultry, soybeans also serve as a key component of human diets, available in various forms. These include direct consumption, such as natto and edamame, as well as processed products like soybean milk and tofu. The development of food-grade soybean varieties has gained increasing attention in recent years, and modern breeding efforts must be aligned with genomic advancements for these varieties—a focus that has been relatively overlooked.

In this research, we included food-grade soybeans such as Zizuka (natto), V10-3653 (soy milk and tofu-producing variety), MFS-561 (sprout variety), and vegetable soybeans (edamame) like VT-Sweet and V16-0565 9-12. These genotypes exhibit varying levels of seed composition (e.g., sucrose and protein) and distinct seed weights (**Figure 1**, **Supplementary Table S1**).

From a consumer perspective, small-seed natto varieties are preferred, while larger edamame seeds are more favored in the market, representing different breeding objectives. In this study, we focused on natto and edamame varieties, aiming to characterize the genomic factors underlying their varied seed sizes. We identified a 319 bp deletion in the promoter region (-448 bp to TSS) of the *MAT1* gene in two food-grade soybean varieties: V10-3653 (soy milk/tofu variety) and V16-0524 (VT-Sweet, an edamame variety) (**Figure 4A**). This deletion resulted in higher expression of *MAT1* in the seeds of these two varieties compared to WM82 (**Figure 4D**). Consistently, the seed weights of these lines were also significantly higher than those of WM82 (**Figure 4E**). However, this deletion was not observed in other sequenced edamame varieties, indicating the complex genomics underlying seed weight. Several genes, including GmST05 and GmSWEET10a, as well as multiple QTLs, have been reported to be associated with seed size and weight (Wang et al., 2020; Zhang et al., 2020; Duan et al., 2022; Luo et al., 2022). However, to date, no SVs have been reported to influence this critical developmental and agronomic trait. The complexity observed in this study may also reflect distinct breeding efforts, as the sequenced natto varieties primarily originate from Japan, while the edamame varieties are mainly adapted to the mid-Atlantic region of the United States. The regulating molecular mechanism through the present deletion in the *MAT1* promoter region will be further investigated.

In the current soybean industry, soybean meal, which contains most of the protein, is primarily used for animal feed, while the oil is largely used in the food industry for human consumption. As protein is a key component of modern human diets, rapid population growth has raised concerns about protein deficiency and the environmental impact of animal-based protein sources. Enhancing plant protein content is therefore crucial for improving both human health and sustainability. In this study, we selected high-protein soybean varieties (V12-4590 and S17-17168) for sequencing and identified 9226 structural variations in these varieties. The newly obtained data, along with further comparative genomics analysis and experimental validation, will help uncover the genetic components and underlying mechanisms associated with protein content and quality in soybean seeds.

In conclusion, by integrating ONT to re-sequence 29 genotypes of grain type soybean and edamame, we identified novel structure variations that have the potential to reveal the basic genetic mechanism associating with important agronomic traits in soybean. It is fundamental to uncover the mechanism underlying these complex traits. These SVs can be also applied to molecular breeding via CRISPR/Cas9-mediated gene editing and to develop markers for soybean breeding selections. Our results provide a new pipeline for understanding basic genetics and rapid marker discovery in crop genomes using structural variation and whole genome sequencing.

## Data Availability

The datasets presented in this study can be found in online repositories. The names of the repository/repositories and accession number(s) can be found in the article/**Supplementary Material**.

## Supplementary material

Supplementary Figure 1 Original agarose gel images corresponding to **Figure 3C** **(A)**, **Figure 4C** **(B)**, and **Figure 5C** **(C)**. The targeted amplicon bands used in this study are labeled with numbers.
