# Supplementary figures and images for: Long-read sequencing reveals novel structural variation markers for key agronomic and quality traits of food-grade soybean
Source: Front Plant Sci. 2025 Apr 8;16:1557748. doi: 10.3389/fpls.2025.1557748 (PMC12011826; doi:10.3389/fpls.2025.1557748)

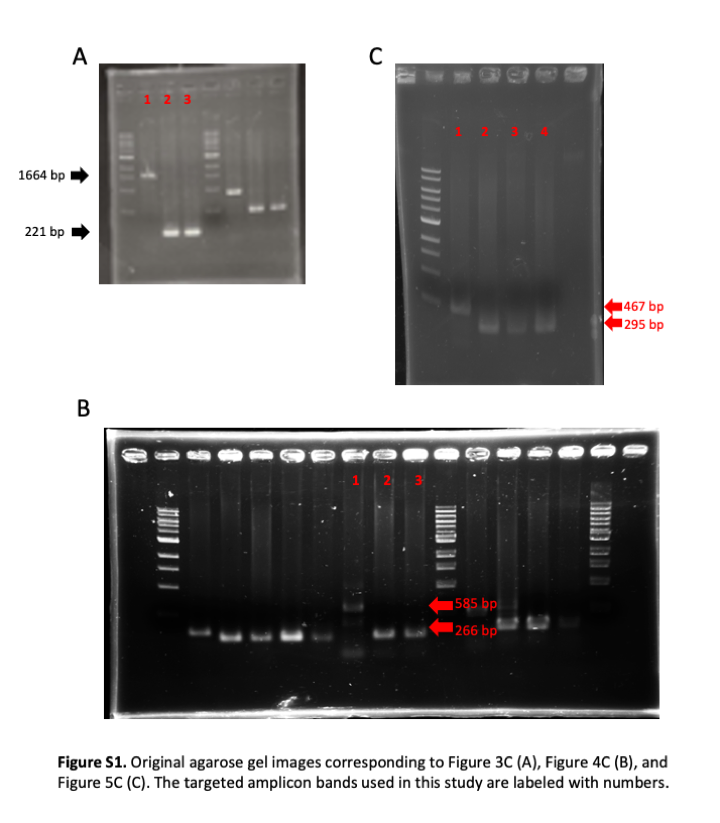

Supplement: Supplementary Figure 1 — Original agarose gel images corresponding to Figure 3C (A), Figure 4C (B), and Figure 5C (C). The targeted amplicon bands used in this study are labeled with numbers. [file Image1.tiff]
